# Single-stage repair of adult aortic coarctation and concomitant cardiovascular pathologies: a new alternative surgical approach

**DOI:** 10.1186/1749-8090-1-18

**Published:** 2006-06-27

**Authors:** Mert Yilmaz, Bulent Polat, Davit Saba

**Affiliations:** 1 Cardiovascular Surgery Department, Faculty of Medicine, Uludag University, Bursa, Turkey; 2 Department of Cardiovascular Surgery Florence Nightingale Hospital, Istanbul Bilim University, Istanbul, Turkey

## Abstract

**Background:**

Coarctation of the aorta in the adulthood is sometimes associated with additional cardiovascular pathologies that require intervention. Ideal approach in such patients is uncertain. Anatomic left-sided short aortic bypass from the arcus aorta to descending aorta via median sternotomy allows simultaneuos repair of both complex aortic coarctation and concomitant cardiac operation.

**Materials:**

Four adult patients were underwent Anatomic left-sided short aortic bypass operation for complex aortic coarctation through median sternotomy using deep hypothermic circulatory arrest. Concomitant cardiac operations were Bentall procedure for annuloaortic ectasia in one patient, coronary artery bypass grafting for three vessel disease in two patient, and patch closure of ventricular septal defect in one patient.

**Results:**

All patients survived the operation and were alive with patent bypass at a mean follow-up of 36 months. No graft-related complications occurred, and there were no instances of stroke or paraplegia.

**Conclusion:**

We conclude that single-stage repair of adult aortic coarctation with concomitant cardiovascular lesions can be performed safely using this newest technique.

## Background

Adult patients with aortic coarctation (CoA) and concomitant cardiac surgically correctable lesions is still dilemma for the surgeons. The optimal operative approach for such patients remains unsettled. Different surgical strategies have been described. One approach is to perform the CoA operation and the additional cardiovascular operation as staged procedures. Situations exist for which one can present a rationale for either operative procedure being the initial operation. Alternative surgical option is single-stage repair of the combined patholgy via median sternotomy. We performed the single-stage operations with Anatomic left-sided short aortic bypass (ALSAB) between arcus aorta and descending aorta using a dacron conduit (6–8 cm) while correcting the other cardiac pathologies simultaneously through the median sternotomy.

## Materials and methods

### Patient 1

**Figure 1:**
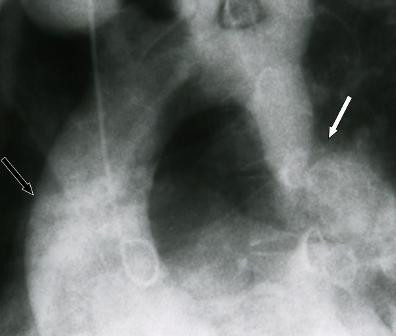
Angiographic appearance of aortic CoA (white arrow) and ascending aortic aneurysm (white line arrow) in case 1.

**Figure 2:**
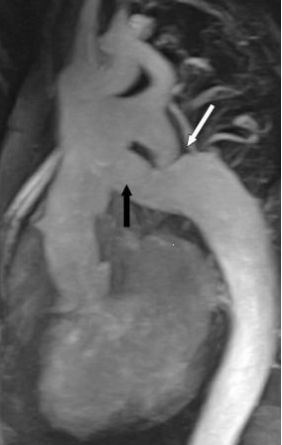
MRA demonstration of aortic CoA (white arrow) and ALSAB graft (black arrow) following the Bentall + ALSAB operation in case 1.

A 27-year-old male presented with congestive heart failure symptoms. Angiography (Figure 1) and echocardiography demonstrated severe aortic CoA and additional annuloaortic ectasia (7 cm Diameter) associated with third degree aortic valve regurgitation. Severe cardiomegaly and poor left ventricular function were also noted (ejection fraction: 27%). Bentall procedure (no. 23 St. Jude Medical^® ^metallic composite Aortic valve (St. Paul, MN, USA)) was performed and the second 22-mm Dacron graft (Hemashield^®^, Boston Scientific Corporation; Natick, Mass) was anastomosed anatomically between the arcus aorta and the descending aorta. Postoperatively, he made a good recovery and was discharged on the 10^th ^postoperative day. Follow-up echocardiography and Magnetic Resonance Angiography (MRA) (Figure 2) showed no evidence of ALSAB graft kinking or compression.

### Patient 2

A 56-year-old male patient presented with unstable angina pectoris and uncontrolable hipertension. Angiography showed CoA of the aorta and three vessel disease with left ventricle hypertrophy. Coronary artery bypass grafting (CABG) (saphenuos vein grafts to the right descending posterior and obtuse marginal branches and left internal mammary artery (LIMA) to the left anterior descending artery) was performed and a 22-mm Dacron graft (Hemashield^®^) was bypassed between the arcus and descending aorta with the same technique. The patient was discharged on the postoperative ninth day without any complication. Patient was in stable condition with adequate function of both ventricules and a substantial decrease in left ventricular hypertrophy as assessed by echocardiography. It also revealed a good functioning graft.

### Patient 3

A 50-year-old male patient was admitted to hospital with unstable angina and uncontrolable hipertansion. Angiographic examination revealed CoA of the aorta and tree vessels disease similar as the previous case. CABG (saphenuos vein grafts to the right coronary artery, obtuse marginal branches and left anterior descending artery) was performed and the 22-mm Dacron graft (Hemashield^®^) graft was anastomossed between the arcus and descending aorta using the same technique. LIMA had atheromatous plaques and because of that reason LIMA was not used. The patient was discharged on the postoperative theerteenth day. Eighteen months later, the patient was asymptomatic and MRA revealed well shaped ALSAB graft.

### Patient 4

A membranous ventricular septal defect (VSD) and serious CoA of the aorta were diagnosed on a 16-year-old boy by echocardiography. VSD was closed using dacron patch and the 16-mm dacron graft (Haemoshield^®^) graft was interpositioned anatomically between the arcus aorta and the descending aorta as in the previous cases. Echocardiography revealed that there was no residual VSD and no graft kinking or gradient between arcus and descending aorta. In postoperative angiography, there was no ALSAB graft kinking or any graft related problem. The patient went on to make straightforward recovery, being discharged on the eleventh postoperative day. Two years following surgery, he was in NYHA class I.

### Surgical technique

Surgery is performed via standard median sternotomy. Cardiopulmonary bypass (CPB) was instituted using right atrial and ascending aortic cannulation. Systemic deep hypothermia (18°C) is used and antegrade and retrograde cardioplegia were administered for myocardial protection. In our patients, concomitant cardiac operations were performed during cooling period. Following these procedures the heart was retracted superiorly, and the posterior pericardium was incised exposing the descending thoracic aorta. During deep hypothermic circulatory arrest (HCA) period, the dacron grafts (Haemoshield) were anastomosed to the descending thoracic aorta end-to-side fashion without using any clamp. The graft was clamped and CPB was started for checking whether there was any leakage from anastomosis or not. Proximal anastomosis was fashioned end-to-side to the internal side of the arcus aorta without using any clamp in another short period of HCA. In patient who required CABG, the LAD-LIMA distal and saphenuos vein grafts proximal anastomosis were performed during rewarming period. CPB was discontinued with no residual gradient between ascending aorta and the descending aorta. Hemodynamic stability of the patients were obtained by adrenaline and noradrenaline infusion during operation and the first two days of postoperative period.

## Results

There were no early or late deaths. None of the patient required reoperation or excesive blood or blood product transfusion. Total HCA times were between 20–24 minutes and no patient presented any neurologic deficit. Although femoral canulation was not use, there was no abdominal organ problem or spinal cord ischemia and also left phrenic or left recurrent laryngeal nerve damage, and chylothorax were not seen. Lengths of ICU stays of the patients were less than 48 hours. All patients survived and were discharged home in good condition. All patients had postoperative control echocardiography and case 1 and 3, 4 had control angiography. None of them showed graft kinking or compression of the tissues. Left ventricular hypertrophy regressed in all patients. None of the patient readdmitted to hospital because of the late complication.

## Discussion

CoA of the descending thoracic aorta generally presents in childhood. The aortic CoA in adult patients is extremely rare; only a few cases where it is the sole congenital malformation or where it is combined with other defects in the same patient have been reported.

Some authors have suggested single-stage procedure in reccurent CoA associated with intracardiac pathologies. [1-3] Vijayanagar et al. were the first to describe performing concomitant aortic valve replacement and the ascending aorta-descending aorta bypass through the posterior pericardium and placing the graft arround the left margin of the heart entirely through a sternotomy incision.[3] Barron et al. have defined two different extra-anatomic bypass techniques.[4]

Pethig et al. pointed out severe hemodynamic instability after relief of the aortic CoA with ascending-descending aorta bypass.[5] They thought that the hypertrophied left ventricle had adapted to high perfusion pressures, relief of isthmic stenosis resulted in a major drop in the ascending aorta postoperatively and this blood pressure appears to be inadequate to maintain sufficient myocardial pressure in hypertrophied left venricules. The large conduit and peripheral vasodilatation may cause a rapid runoff and resultant coronary steal immediately after discontuning CPB circulation. For that reason, weaning from bypass should be under adrenalin and noradrenaline infusion in that kind of patients. Mulay et al. reported three patients, the intracardiac pathologic lesions were corrected first, and the CoA was repaired as a second-stage procedure 6 weeks later.[6] They defined that the single-stage approach would have caused a sudden decrease in systemic vascular resistance during coming off bypass and that could be the reason of hemodynamic instability as Pethig mentioned in their article.

In our experience, the most crucial point was the afterload management during weaning from bypass. We believe that carefull adrenaline + noradrenaline infusion is enough to provide sufficient peripheral vascular resistance. The use of CPB also adds safety for patients with unstable hemodynamics. Operating on the cardiac defect without addressing the significant CoA may lead to significant hypoperfusion of organs distal to CoA and severe afterload increase may stress the left ventricle causing pump failure. [7,8]

Any attempts at CoA repair in these patients would be disastrous without prior or simultaneous coronary revascularisation. The internal mammary arteries are often increased in size and are unsuitable for use as conduit for revascularization.[9] In patients requiring coronary artery bypass grafting in combination with CoA repair, care must be taken to ensure adequate mammary artery flow before its use, because of its greater susceptibility for atherosclerotic narrowing. LIMA graft was used only in one patient that required CABG, in our two cases.

Fedoruk et. al. reported compression of esophagus causing dysphagia in a 9 year-old child due to the extra-anatomic bypass is lying on the right side of the heart.[10] In this route, the graft length is at least 2 times longer than the left route. Additionally, the graft is passing from above or behind the inferior vena cavae and lying arround the right atrium which could have risk for compression of surrounding tissues but this statement is not declared clearly by the authors.

The mortality and morbidity of a staged surgical approach is significant, irrespective of the sequence of repair. Correction of the coarctation alone is associated with increased perioperative myocardial infarction.[5] On the other hand, correction of the cardiac lesion alone is associated with increased postoperative renal failure and paraplegia as a result of inadequate perfusion distal organ perfusion.[11] In adulthood, the dependency of the spinal cord blood supply on fewer radicular arteries increases the risk of paraplegia developing during the postoperative period. The technique of hypothermic CPB with HCA has several advantages when applied to adult patients with complex forms of CoA. It facilitates adequate exposure of the structures involved, avoids placement of clamps on fragile tissue, and provides adequate protection of the brain, the spinal cord, and other organs.

The main indications for single-stage repair are:

1. Calcified or serious adult CoA with concomitant cardiovascular pathologies required surgery.

2. CoA with serious triple coronary artery disease.

3. Re-CoA with concomitant cardiovascular pathologies required surgery.

For the treatment of CoA and associated with cardiac anomalies, we have utilized the use of ALSAB between the arcus aorta and the descending aorta without side-biting clamp under HCA which was never used or published before. Side-biting clamp using in the hypertansive patients and mostly atherosclerotic aorta has a risk of neurologic complications and also neighbour organ (esophagus) and collateral artery damage at the distal anastomotic side could be expected dispate the all reporters not noticed any that kind of problem so far. The limitation of using our technique is extensive calcification at the arcus aorta. Hypothermic CPB and HCA techniques lend a margin of a safety for spinal cord ischemia.[8,12] We believe that the use of CPB is the best method and HCA provides a very dry field for surgeon to perform aortic anastomosis and also reduces the risk of paraplegia.

Our technique's superiority against the previous methods is single incision, short graft length and not using/no necessity side-biting clamp that could be the reason of neurologic disorder or neighbour organ damage such as esophagus. The use of HCA has some risks but it has provided easy exposure of the distal thoracic aorta and avoided the necessity of side-biting clamp. Even though some authors suggested different extra-anatomic routes for the bypass conduit, ALSAB technique might reduce the risk of kinking and long graft requirement. We conclude that single-stage repair of CoA and associated cardiovascular lesions can be performed safely and effectively using this technique without the risk of graft related problems.
